# The monitoring of B lymphocytes in non-lymphoma patients following rituximab treatment

**DOI:** 10.3389/fimmu.2024.1513303

**Published:** 2024-11-25

**Authors:** Linjie Dong, Lin Yan, Yi Li, Mei Li, Weihua Feng, Xiaoqiong Li, Jiaxi Yue, Erdi Zhang, Yao Luo, Yangjuan Bai

**Affiliations:** ^1^ Department of Laboratory Medicine, West China Hospital, Sichuan University, Chengdu, Sichuan, China; ^2^ Department of Laboratory Medicine, Meishan City People’s Hospital, Meishan, Sichuan, China

**Keywords:** rituximab, membranous nephropathy, kidney transplantation, neuromyelitis optica, pemphigus, B lymphocytes

## Abstract

RTX was initially used for non-Hodgkin’s lymphoma treatment and has been used in the clinical treatment of various autoimmune diseases as well as in antirejection and immune induction therapy for kidney transplant recipients. Following RTX treatment, the time for B cell regeneration varies among patients, but there is no unified recommendation for the frequency of B cell monitoring. This study aimed to investigate the clinical significance of periodic monitoring of peripheral blood B lymphocytes in individualized immunotherapy following rituximab (RTX) treatment in patients with different diseases. This study included 488 patients with different diseases divided in four groups who were hospitalized and followed up from April 2017 to March 2024 (including 77, 161, 120, and 130 cases of neuromyelitis optica, pemphigus, membranous nephropathy, and kidney transplant recipients, respectively). Dynamic changes in percentage and absolute count of peripheral blood B lymphocytes before and after RTX treatment were investigated in the four groups, as well as the number of B cell subsets in 32 patients with optic neuromyelitis after RTX treatment. Although most patients showed high expression of B cells after 24 weeks, less than 6.8% of patients still began to experience B cell regeneration within 4 weeks. Thus, regular B cell monitoring following RTX treatment is helpful to better track the remission and recurrence of the disease and provide effective laboratory support for the selection and implementation of individualized immunotherapy.

## 1 Introduction

Personalized medicine has been extensively discussed in the context of cancer treatment. Patients usually show variable responses to the same therapy, suggesting heterogeneity of patients and the need for more precise therapies. Many studies have demonstrated that the development, hyperactivation, and differentiation of B cells are critically regulated by epigenetic modifications (1), which may affect the disruption of immune tolerance and the progression of autoimmune diseases. Hyperactivation of B cells with massive production of auto antibodies are hallmark features of many autoimmune diseases, which highlights the crucial role of B cells in the pathogenesis of autoimmune diseases.

CD20 is a specific labeled antigen on the surface of B cells, expressed during early and mature stage B cells (2). It is also expressed on B cell-derived malignant tumor cells; however, CD20 is not expressed on progenitor B cells and plasma cells. Therefore, CD20 has become a therapeutic target for B cell malignant tumors and autoimmune diseases (3). Rituximab (RTX) is a human–mouse chimeric monoclonal antibody targeting CD20 (4) that can effectively antagonize B cells, was initially used for non-Hodgkin’s lymphoma treatment (5). RTX specifically binds to CD20 and eliminates CD20+ B cells through various mechanisms, including antibody or complement-dependent cytotoxicity, antiproliferative effect, antibody-dependent cellular phagocytosis, or apoptosis induction, thereby inhibiting pathogenic antibody generation (6). RTX has been used in the clinical treatment of various autoimmune diseases as well as in antirejection and immune induction therapy for kidney transplant recipients.

The most effective treatment for end-stage kidney disease is renal transplantation; however, acute rejection (AR) can significantly reduce the survival rate renal transplantation patients and can even be fatal (7). The use of RTX for preoperative immune induction therapy and postoperative antirejection therapy can effectively prevent and control AR (8). With the discovery of anti-PLA2R and anti-THSD7A antibodies in patients with membranous nephropathy (MN), a new concept of MN as an autoimmune disease was propose (9). Ruggenti et al. used RTX to treat 50 patients with idiopathic MN. Comparative observations revealed that RTX can effectively reverse renal pathological changes and delay disease progression (10). Pemphigus is a relatively rare autoimmune skin disease that involves the skin and mucosa and its pathogenesis has not been fully elucidated. Autoantibodies targeting desmoglein 1 and desmoglein 3 play a significant role in the occurrence and development of pemphigus (11). Neuromyelitis optica (NMO) and NMO spectrum disorders are central nervous system autoimmune diseases mediated by aquaporin 4 antibodies (AQP4-IgG) that can simultaneously or sequentially affect the optic nerve and spinal cord (12). The B cells in the patient’s body are initially activated and synthesized to secrete AQP4-IgG in the periphery (13). Subsequently, AQP4-IgG enters the central nervous system through the blood–brain barrier, leading to optic neuromyelitis spectrum diseases (14). B cell-targeted therapy can help control and alleviate diseases by reducing B cell levels and circulating autoantibody production. As CD20 is not expressed in hematopoietic stem and progenitor B cells, the body can still rebuild the B cell population after stopping anti-CD20 therapy (3). The regeneration of B cells and the reproduction of pathogenic antibodies lead to tumor recurrence, rapid disease progression or rejection reactions. The timely detection of B cell repopulation and subsequent RTX treatment are crucial for consolidating therapeutic efficacy and improving patient prognosis.

**Figure 1:**
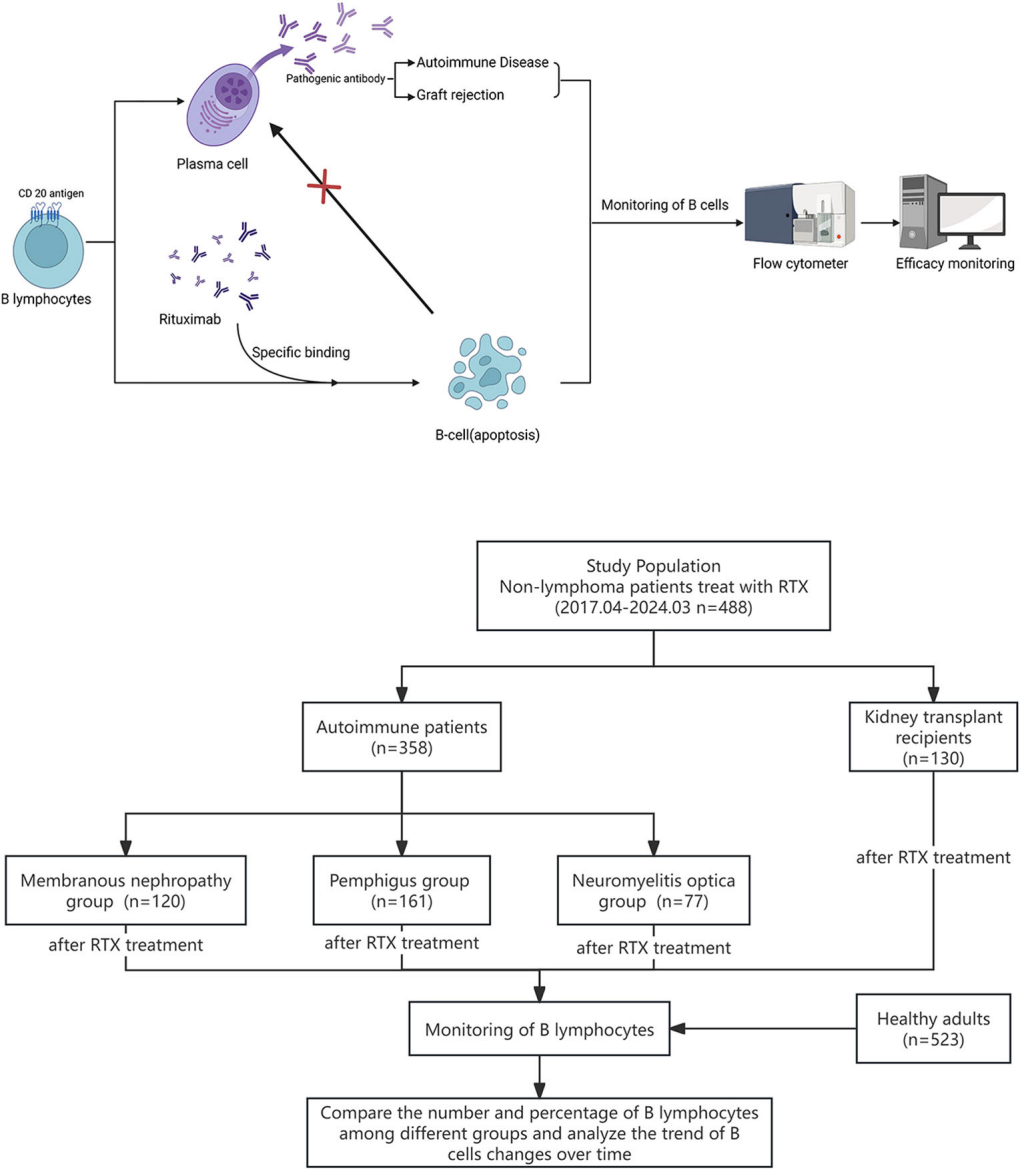
Graphic about the experimental design and research flowchart for the monitoring of B cells in patients after RTX treatment.

Recent studies have revealed that epigenetic dysregulation may lead to aberrant expansion of pathogenic B-cell subsets during the development of autoimmune diseases (15). Therefore, as the target cells for RTX-targeted clearance, the effective monitoring of peripheral blood B cell can provide a basis for individualized RTX treatment. This study aimed to explore the clinical significance of regular peripheral blood B cell monitoring in RTX individualized treatment in patients with different types of non-lymphoma diseases. The research process is shown in **Figure 1**.

## 2 Materials and methods

### 2.1 Detection of peripheral blood B cells and their subsets

Heparin anticoagulant peripheral blood samples were collected from the enrolled participants before RTX treatment, within 4 weeks after treatment, as well as at 8, 12, 16, 24, 36 weeks after treatment and up to one year after treatment. Mixed anticoagulated whole blood with fluorescently labeled monoclonal antibodies targeting CD45 (PerCP, BD Company, USA), CD3 (APC, BD Company, USA), and CD19 (PE, BD Company, USA) for specific reactions. Subsequently, to detect labeled lymphocytes, hemolysis and BD FACSCAntoII flow cytometry was used. Subsequently, the fluorescence signal obtained by irradiating lymphocytes with a laser source was converted into image and digital signals by a computer to obtain the percentage of CD19+ CD3- lymphoid cells in CD45+ lymphocytes, which was the percentage of peripheral blood B cells. Calculate the absolute count of B cells using a BD Trucount Tube (BD Company, USA) containing a known number of beads using a formula (cells/μL = obtained cell count × total beads/[beads obtained × sample size]).

Using the same method, anticoagulated whole blood was specifically reacted with fluorescently labeled anti-CD45(KRO, Beckman Coulter Company, USA) CD3(FITC, Beckman Coulter Company, USA), CD14(APC, Beckman Coulter Company, USA), CD19(PE, Beckman Coulter Company, USA), CD27(PC7, Beckman Coulter Company, USA), and CD38(APC-A750, Beckman Coulter Company, USA) monoclonal antibodies. After hemolysis, the labeled individual lymphocytes were measured using a DXFLEX flow cytometer (Beckman Coulter Company, USA). CD3-CD14-CD19+CD27- cells are identified in B lymphocytes, referring to the population of naive B cells, the subset of CD3-CD14-CD19+CD27+ cells represent memory B cells, while the CD3-CD14-CD27highCD38high population corresponds to plasmablast.

### 2.2 Statistical analysis

Statistical Package for the Social Sciences (version 22, IBM, Armonk, NY, USA) and GraphPad Prism 9.0 (GraphPad software, USA) were used for statistical analysis and plotting. Moreover, nonparametric tests were used for intergroup comparisons. *p* < 0.05 indicated a statistically significant difference.

## 3 Results

### 3.1 Study participants characteristics

A retrospective analysis was conducted on 488 patients with non-lymphoma who underwent RTX treatment and follow-up at the West China Hospital of Sichuan University from April 2017 to March 2024, and compared with 523 healthy adults. Among them, there were 130 KT recipients (male/female, 96/34; age, 18–62 years, 44 postoperative anti-rejection treatments and 86 pretransplant induction treatments, including 5 ABO blood group incompatible recipients), 120 patients with MN (male/female, 80/40; age, approximately 19–79 years), 161 patients with pemphigus (male/female, 68/93; age, 17–78 years old), and 77 patients with NMO (male/female, 8/69; age, 17–70 years) were categorized as the kidney transplant, MN, pemphigus, and optic neuromyelitis groups, respectively. Inclusion Criteria: 1. KT recipients who received RTX induction treatment before surgery, KT recipients who received RTX postoperative anti-rejection treatments; patients clinically diagnosed with MN who received RTX treatment; patients clinically diagnosed with pemphigus who received RTX treatment; and patients clinically diagnosed with NMO who received RTX treatment; 2. regular follow-up in our hospital for more than 24 weeks; 3. the percentage and absolute count of peripheral blood B lymphocytes tests have been performed more than twice within 24 weeks after RTX treatment. Differences in the use of RTX were observed in each group, as shown in **Table 1**. This study was approved by the ethics committee of West China Hospital. Informed consent was obtained from all individual participants included in the study.

**Table 1:** Gender, age, and partial dosage of RTX treatment in non-lymphoma patients.

Group	Gender		Age of first treatment			Dosage of RTX (mg)						
	male	female	17~44	45~59	60~89	100	200	300	400	500	600	700
KT group: ABOi-KT	3	2	5				4					
ABOc-KT	58	23	76	5		2	18	3				
Postoperative AR	35	9	29	14	1	5	5	1		8		
MN group	80	40	39	55	26	21	10			5	1	1
Pemphigus group	68	93	60	67	34	13				53	14	
NMO group	8	69	41	27	9			1	2	7		

KT, kidney transplant; MN, membranous nephropathy; NMO, neuromyelitis optica; ABOi-KT, ABO-incompatible kidney transplant; ABOc-KT, ABO-compatible kidney transplant.

### 3.2 Analysis of the number and percentage of peripheral blood B cells prior to RTX treatment and at the first follow-up following RTX treatment

**Figure 2:**
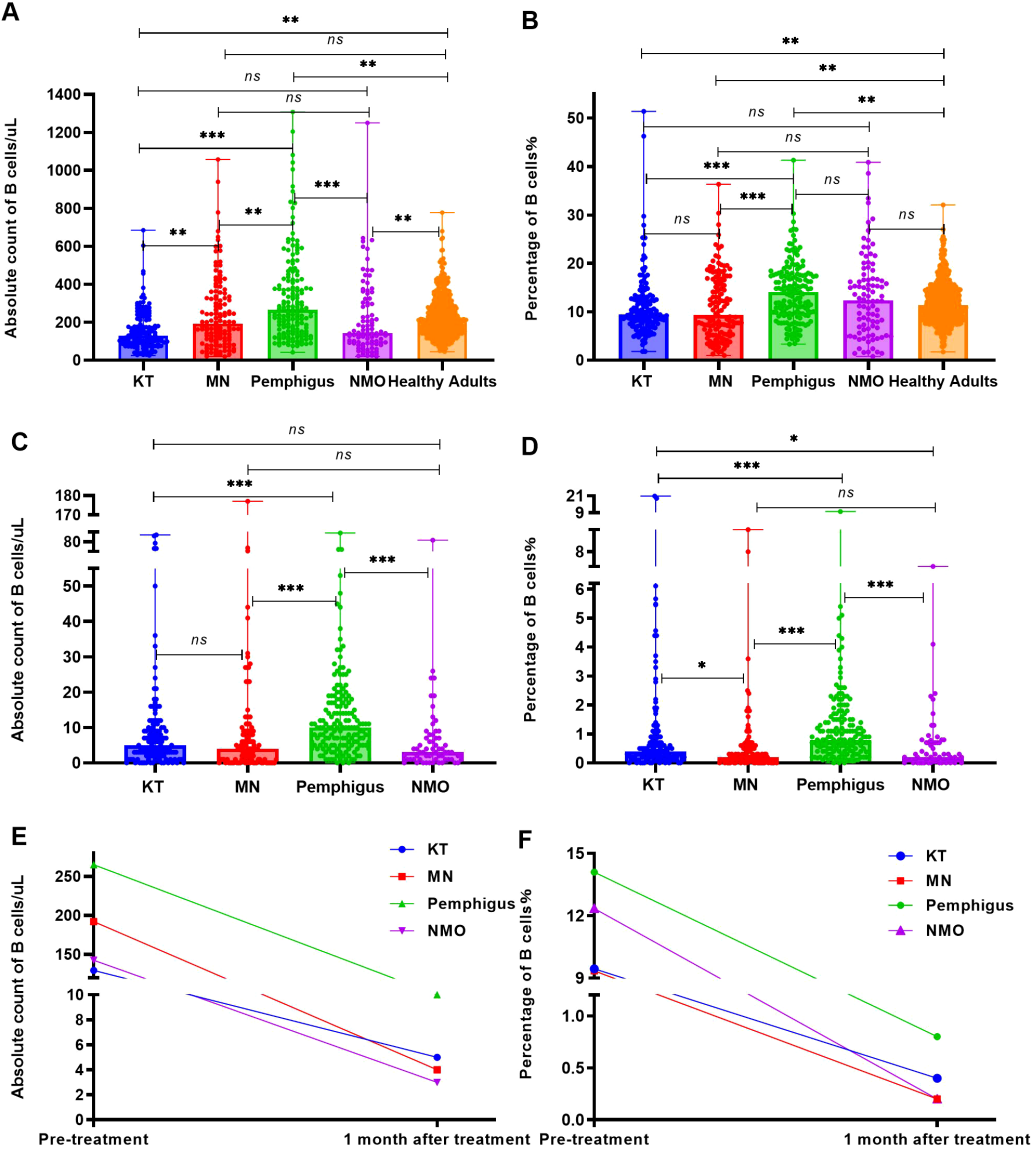
The number and percentage of B cells before and one month following RTX treatment. **(A)** Analyze and compare the number of peripheral blood B cells in the four groups of patients and healthy adults before RTX treatment. **(B)** Analyze and compare the percentage of peripheral blood B cells in the four groups of patients and healthy adults before RTX treatment. **(C)** Analyze and compare the number of peripheral blood B cells in the four groups of patients within one month following RTX treatment. **(D)** Analyze and compare the percentage of B cells in the four groups of patients within one month following RTX treatment. **(E)** Changes in the absolute count of peripheral blood B cells in all four groups before and after RTX treatment. **(F)** Changes in the percentage of peripheral blood B cells in all four groups before and after RTX treatment. "*", *p*<0.05; “**”, *p* < 0.005; “***”, *p* < 0.001; “*ns*”, no significant difference.

Before RTX treatment, the average levels of peripheral blood B cell absolute count in patients with pemphigus (median 265 cells/uL) were the highest, significantly higher than those in the healthy adults (median 209 cells/uL, *p* = 0.005) and patients with KT (median 129.5 cells/uL, *p* < 0.001), MN (median 192 cells/uL, *p* = 0.004) and NMO (median 142.5 cells/uL, *p* < 0.001), as **Figure 2A**. Moreover, the percentage of B cells in patients with pemphigus (median 14.1%) were significantly higher than those in the healthy adults (median 11.43%, *p* = 0.002) and patients with KT (median 9.45%, *p* < 0.001), MN (median 9.35%, *p* < 0.001), but no significant difference was observed between the pemphigus group and NMO group (median 12.35%, *p* > 0.05). The results are depicted in **Figure 2B**.

Within one month following RTX treatment, a total of 62 patients(including KT group: 22 cases,16.9%; MN group: 14 cases, 11.6%; Pemphigus group: 1 case, 0.6%; NMO group: 25 cases, 32.4%) did not undergo B cell monitoring, and the number and percentage of peripheral blood B cells in the remaining patients were analyzed during the first follow-up. The absolute count and percentage of peripheral blood B cells in patients with MN (median 4 cells/uL, 0.2%; *p* < 0.001), NMO (median 3 cells/uL, 0.2%; *p* < 0.001), and KT (median 5 cells/uL, 0.4%; *p* < 0.001) were significantly lower than those in the pemphigus group (median 10 cells/uL, 0.8%), as **Figures 2C, D**. The absolute count and percentage of peripheral blood B cells in all four groups were significantly reduced (*p* < 0.001) compared with those before treatment as shown in **Figures 2E, F**.

### 3.3 Analysis of the trend of changes in the number and percentage of B cells over time following RTX treatment

**Figure 3:**
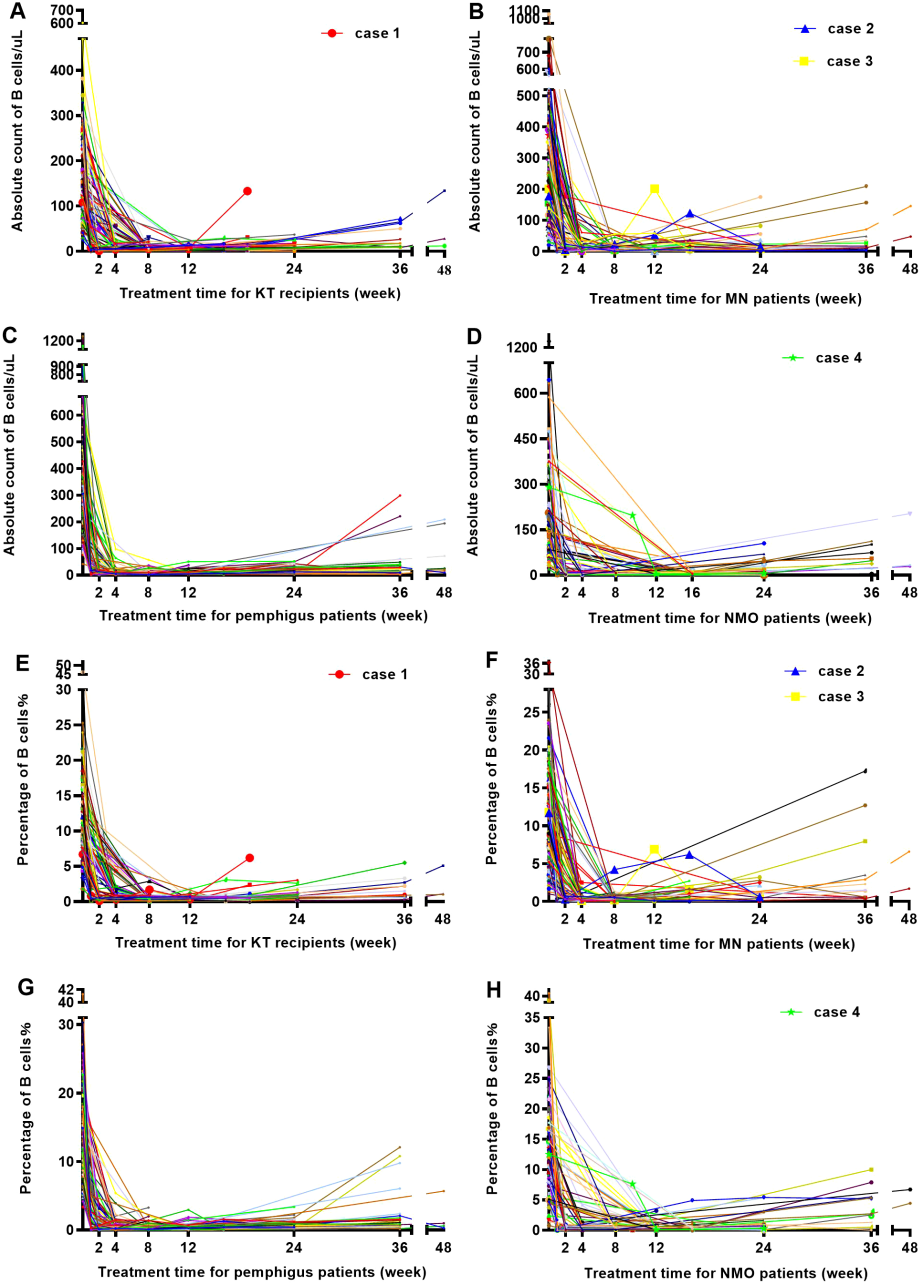
Trends in the counts and the percentages of B cells in the four groups as RTX treatment progressed. **(A)** Changes in B cell counts over time in KT recipients following RTX treatment. **(B)** Changes in B cell counts over time in patients with MN following RTX treatment. **(C)** Changes in B cell counts over time in patients with pemphigus following RTX treatment. **(D)** Changes in B cell counts over time in patients with NMO following RTX treatment. **(E)** Changes in the percentage of B cells over time in KT recipients following RTX treatment; **(F)** Changes in the percentage of B cell over time in patients with MN following RTX treatment. **(G)** Changes in the percentage of B cells over time in patients with pemphigus following RTX treatment. **(H)** Changes in the percentage of B cells over time in patients with NMO following RTX treatment.

Within 24 weeks following RTX treatment, the four groups underwent multiple tests of peripheral blood B cell absolute count and percentage determination. The absolute count (**Figures 3A-D**) and percentage (**Figures 3E-H**) of peripheral blood B cells in most patients significantly decreased and remained low for approximately 4 months following RTX treatment; however, there remained 4 patients (4 in 488 patients) whose absolute count and percentage of B cells showed significant fluctuations following treatment.

Case 1 was a kidney transplant recipient who received induction therapy before surgery. After a single 200 mg RTX infusion, the number of B cells in the patient decreased to 1 cell/μL, and the percentage of B cells was 0.1% following one week of treatment. One month (4 weeks) after receiving RTX treatment, the patient underwent kidney transplantation, at which point the B cell count remained at 1 cell/μL. However, at 20 weeks following RTX treatment (15 weeks following kidney transplantation), the absolute count of B cells increased to 133 cells/μL, as **Figure 3A**, and the percentage of B cells reached 6.2%, as **Figure 3E**.

Case 2 and 3 were patients with MN. At 15 weeks following a single 100 mg RTX infusion, the absolute count of B cells in case 2 increased from 6 cells/μL to 53 cells/μL, and the percentage of B cells increased from 0.3% to 4.2%. At 19 weeks, upon re-admission, the absolute count of B cells increased to 123 cells/μL, and the percentage of B cells increased to 6.2%. Therefore, RTX infusion (200 mg) was performed again. After 4 weeks of the second treatment, the absolute count of B cells decreased to 15 cells/μL, as **Figure 3B**. And the percentage of B cells decreased to 0.6% in **Figure 3F**. Case 3 showed a B cell absolute count of 1 cell/μL and a B cell percentage of 0.1% at the third week following a single 100 mg RTX infusion. After 3 months of treatment, the absolute count of B cells increased to 202 cells/μL and a B cell percentage of 6.9%. The patient was readmitted and treated with RTX infusion before discharge.

Case 4 was a patient with NMO. Case 4 did not undergo B cell testing within one month after receiving a single 400 mg RTX infusion. The first test was conducted 10 weeks following treatment, with an absolute B cell count of 197 cells/μL and a B cell percentage of 7.6%. Within 1 week after receiving another 500 mg RTX infusion, the number of B cells decreased to 4 cells/μL in **Figure 3D**, and the percentage decreased to 0.1% as **Figure 3H**.

### 3.4 Analysis of the number of B cell subsets following RTX treatment

**Figure 4:**
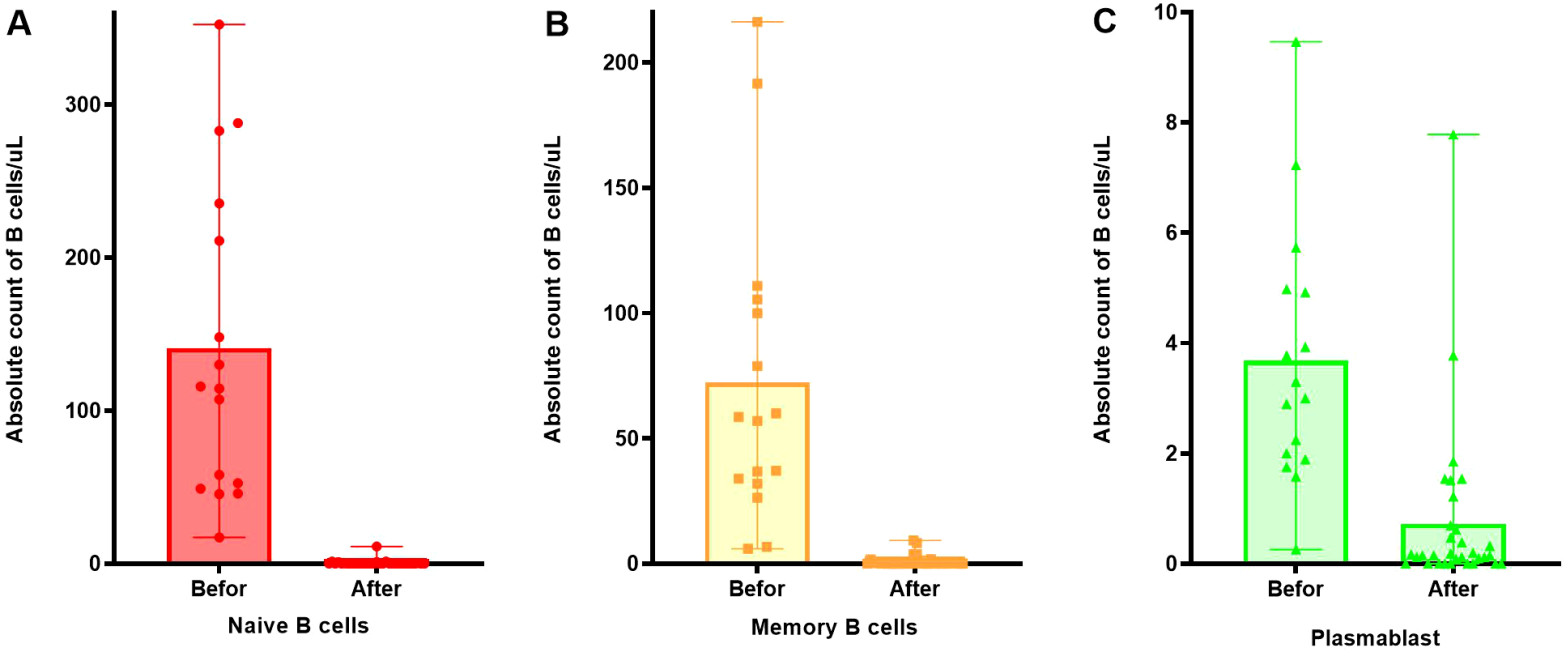
Changes in the number of B cell subsets before and after RTX treatment. **(A)** Changes in the number of naive B cells before and after RTX treatment. **(B)** Changes in the number of memory B cells before and after RTX treatment. **(C)** Changes in the number of plasmablast before and after RTX treatment.

After RTX treatment, naive B cells and memory B cells significantly decreased (*p* < 0.001) in **Figures 4A, B**, however, some plasmablasts still remained at high values, as **Figure 4C**.

### 3.5 Analysis of B cell regeneration within one year following RTX treatment

**Figure 5:**
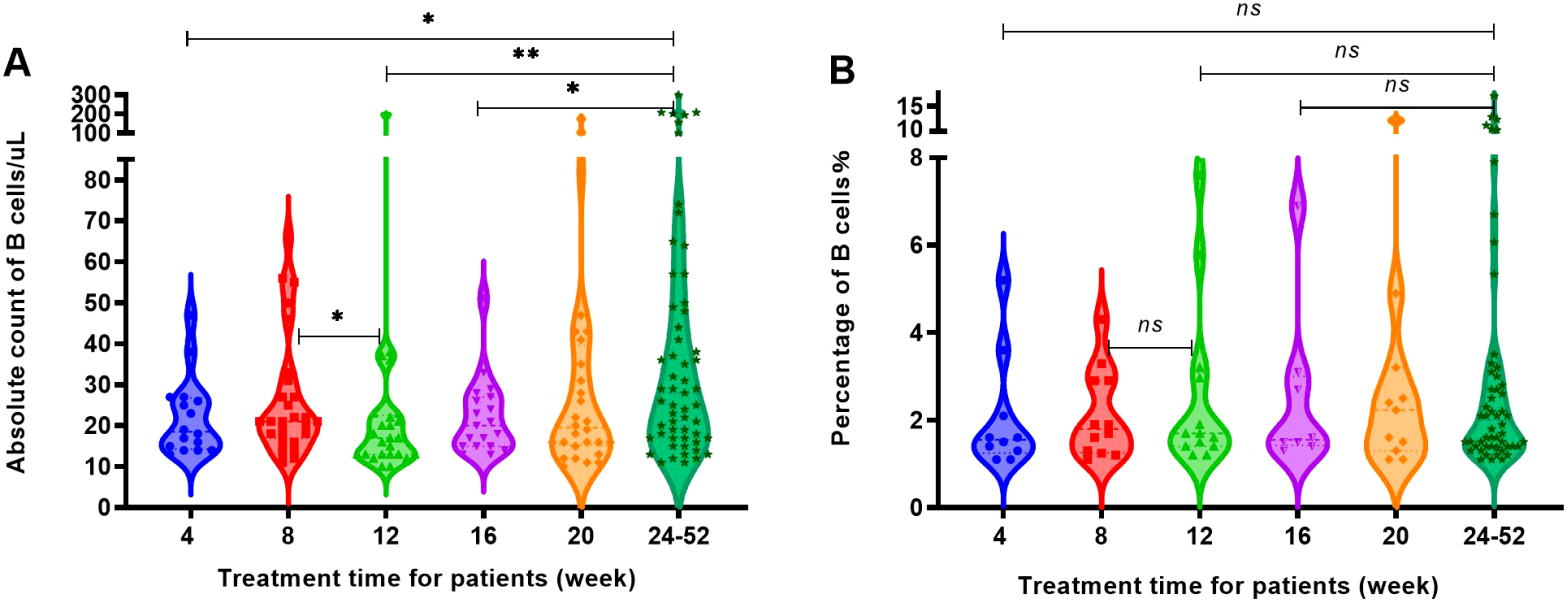
The increase in the number and percentage of B cells in patients with regeneration at different time periods. **(A)** The B cell count of patients with regeneration at different time periods. **(B)** The percentage of B cells in patients with regeneration at different time periods. "*", *p*<0.05; "**", *p*<0.005; "*ns*", no significant difference.

After RTX treatment, four groups of patients were defined for regeneration based on an increase in B cell count exceeding 10 cells/uL and an increase in B cell percentage exceeding 1% of CD45^+^ lymphocytes, respectively. The increase in B cell count at 8 weeks was higher than that at 12 weeks and the increase in B cell count at 4, 12 and 16 weeks were significantly lower than that at 24 weeks, as **Figure 5A**. It could be seen that patients experienced regeneration every month, but most of the regeneration occurred after 24 weeks. The percentage of patients with regeneration occurring after 24 weeks to the total number of regenerated patients were 33.2% for B cell absolute count criteria (**Figure 5A**) and 50% for B cell percentage criteria (**Figure 5B**).

**Figure 6:**
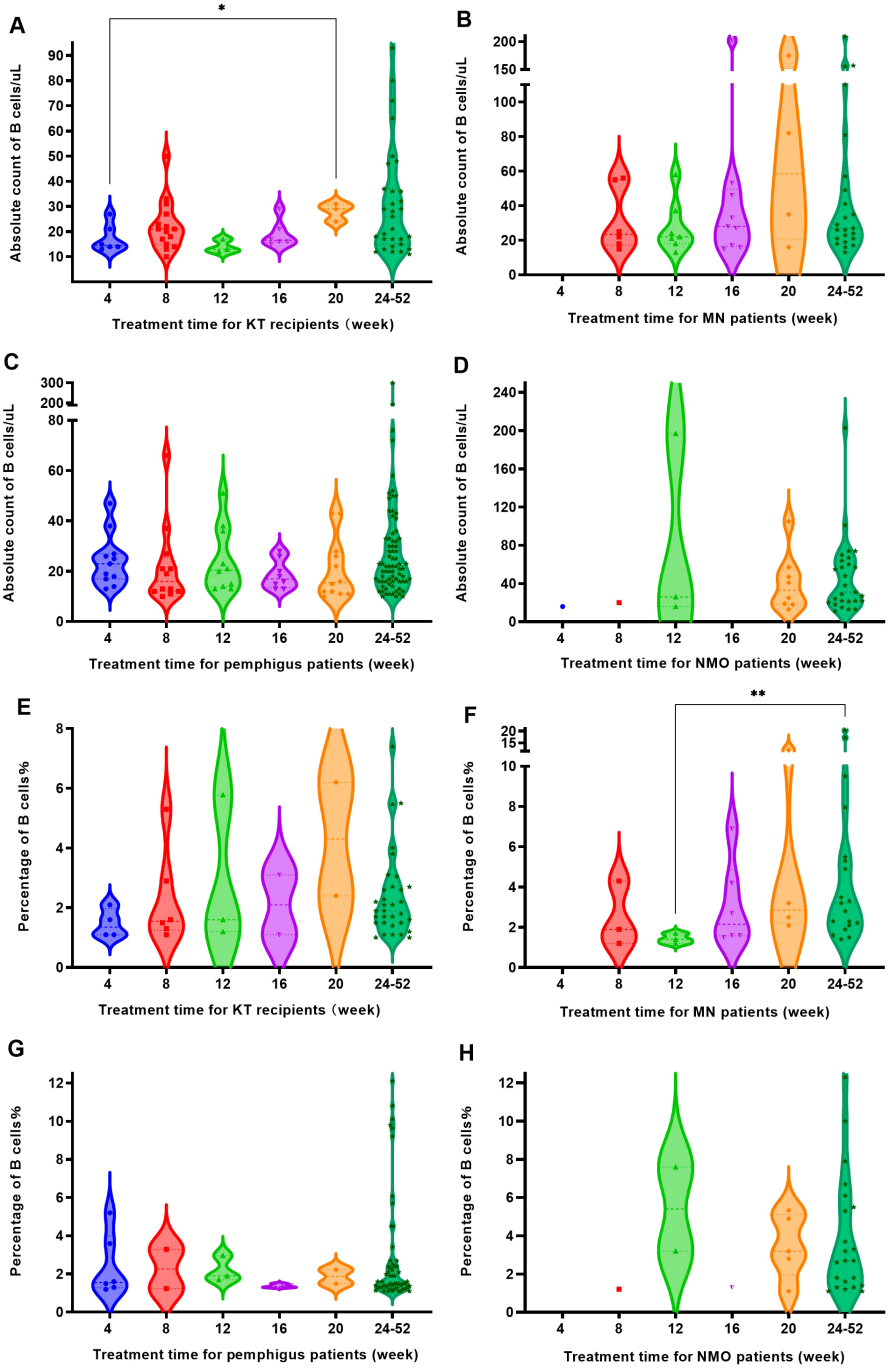
The B cell regeneration observed in four groups of patients at different time periods. **(A)** KT recipients experienced B cell regeneration (with a count increase of over 10 cells/uL) at different time periods. **(B)** MN patients experienced B cell regeneration (with a count increase of over 10 cells/uL) at different time periods. **(C)** Pemphigus patients experienced B cell regeneration (with a count increase of over 10 cells/uL) at different time periods. **(D)** NMO patients experienced B cell regeneration (with a count increase of over 10 cells/uL) at different time periods. **(E)** The regeneration of B cells (with a percentage increase of over 1%) in KT recipients at different times after RTX treatment. **(F)** The regeneration of B cells (with a percentage increase of over 1%) in MN patients at different time points after RTX treatment. **(G)** The regeneration of B cells (with a percentage increase of over 1%) in pemphigus patients at different time points after RTX treatment. **(H)** The regeneration of B cells (with a percentage increase of over 1%) in NMO patients at different time points after RTX treatment. "*", *p*<0.05; "**", *p*<0.005.

B-cell regeneration with an increase of 10 cells/uL or more occurred in all four disease groups. It was found that KT recipients and pemphigus patients experienced regeneration every month, while most MN and NMO patients began to experience regeneration from the 8th week in **Figures 6A-D**. The majority of patients in the four disease groups experienced regeneration after 24 weeks, accounting for 48.5% of the total regeneration in KT recipients, 46.9% of the total regeneration in MN patients, 58.9% of the total regeneration in pemphigus patients, and 67.5% of the total regeneration in NMO patients, respectively.

However, when using the detection of CD19^+^ cell elevation accounts for over 1% of the total number of CD45^+^ lymphocytes as the regeneration criterion, the number of regenerating patients was lower than that defined by an increase in B cell count of more than 10 cells/uL. Similarly, KT recipients and pemphigus patients still experienced regeneration every month, while MN patients and NMO patients began to experience regeneration from the 8th week, as shown in **Figures 6E-H**. After 24 weeks of treatment, the number of regenerated patients in each disease group increased, accounting for 66% of the total regenerated patients in KT recipients, 54.3% of the total regenerated patients with MN, 77% of the total regenerated patients with pemphigus, and 71% of the total regenerated patients with NMO, respectively.

**Figure 7:**
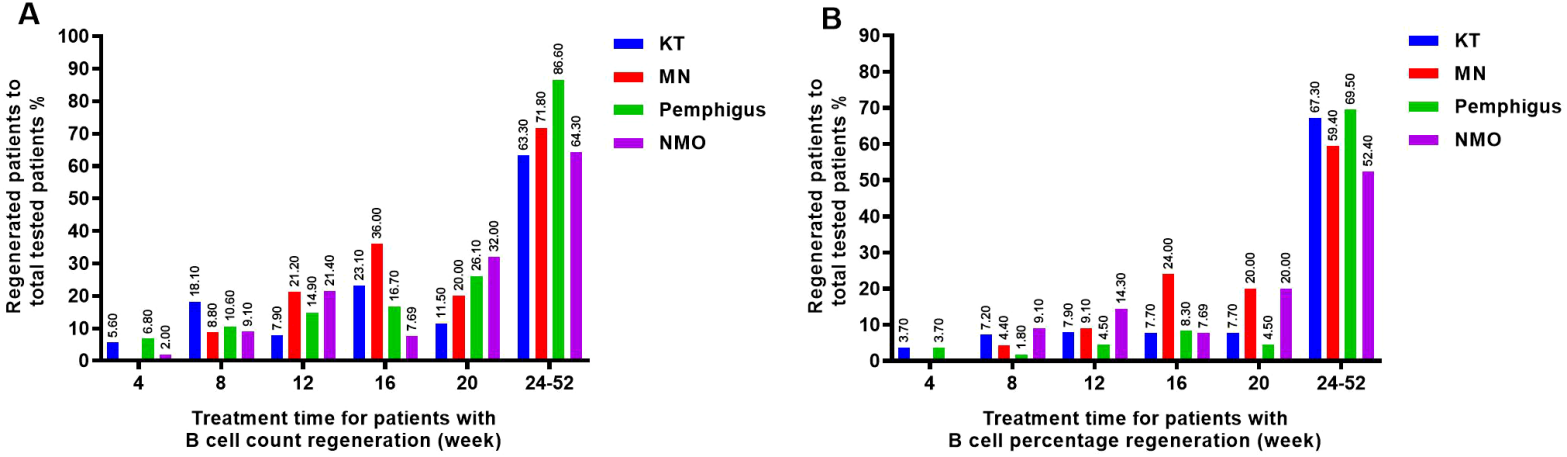
The probability of B cell regeneration in patients with different diseases at different time periods. **(A)** The probability of B cell count increasing by more than 10 cells/uL in patients with different diseases at different time periods. **(B)** The probability of B cell percentage growth exceeding 1% of CD45^+^ lymphocytes in patients with different diseases at different time periods.

Within 8 weeks after treatment, the regeneration rate of KT recipients was relatively high, with the B cell count and percentage regeneration rate of 18.1% and 7.2%, respectively. Between 12 and 20 weeks, the regeneration rate of MN patients was more significant, and at 16 weeks, the regeneration rates of B cell count and percentage were as high as 36% and 24%. After 24 weeks, the regeneration rate of B cell count in most patients exceeded 60%, and the regeneration rate of B cell percentage exceeded 50%. Among them, pemphigus patients had the highest regeneration rate, with a regeneration rate of 86.6% and 69.5% for B cell count and percentage, as **Figure 7**.

## 4 Discussion

RTX, a CD20 monoclonal antibody, is highly efficient in treating autoimmune diseases. It can effectively alleviate proteinuria in patients with MN and reduce the rate of recurrences and adverse events, particularly in patients who have not responded to hormone and other immunosuppressive treatments or have relapsed after remission. Owing to the fact that only about 70-80% of patients with MN and NMO have positive expression of the main pathogenic antibody (16, 17), it is not possible to evaluate disease progression and efficacy monitoring through autoantibody levels for patients with negative expression of pathogenic antibodies, making it difficult to achieve individualized and effective treatment. Thus, complete clearance of peripheral blood B cells can be used for evaluating the therapeutic response and efficacy of CD20 monoclonal antibodies owing to the direct action of RTX on B cells. Moreover, decrease or rebound of peripheral blood B cells typically occurs earlier than changes in pathogenic antibody levels after anti-CD20 therapy, thereby making it an effective means of monitoring RTX treatment and guiding the adjustment of treatment plans.

At present, the following two treatment options for autoimmune diseases and kidney transplant recipients using RTX are available: (1) intravenous infusion of RTX with a body surface area of 375 mg/m^2^, once a week, for four consecutive weeks, and (2) 1,000 mg intravenous infusion, administered twice, at a 2-week interval (16–18). Some patients also receive low-dose administration, with a single intravenous infusion of approximately 500–600 mg or 100 mg, once a week, for 4 weeks. Most patients can maintain a depleted state of B lymphocytes for approximately 6–8 months following treatment (16). Patients generally experience B cell regeneration after 6–12 months (19), at which point RTX treatment needs to be repeated. Some researchers believe that after CD19^+^ cell depletion is detected by flow cytometry, the detection of CD19^+^ cell elevation accounts for over 1% of the total number of CD45^+^ lymphocytes, indicating the occurrence of B cell regeneration (19, 20), suggesting that patients need to enter the next treatment cycle. However, some schemes also use CD19^+^ cells increased by 2% or CD19^+^ cell absolute count increased by 10 cells/μL as the criterion for the next treatment cycle (20, 21). In other studies, it has been reported that after the first dose of RTX administration, the subsequent medication regimen can be optimized by monitoring the levels of peripheral blood B cell subsets (22). Our research is consistent with these studies. It shows that the B cell levels in patients with pemphigus were significantly higher than those in other disease groups and healthy control groups before RTX treatment. After RTX treatment, all four disease groups showed a significant decrease, but pemphigus remained the highest. Meanwhile, some patients still maintained high levels of plasmablasts after RTX treatment. Less than 6.8% of patients experienced a rebound within 4 weeks. After that, although the probability of B cell regeneration increased, the majority of patients’ B cells remained at a relatively stable low level within 20 weeks. More than 64% (with a count increase of over 10 cells/uL) and 76% (with a percentage increase of over 1%) of patients maintained low levels of B cell count and percentage within 20 weeks after RTX treatment. However, the proportion of patients who experienced rebound over time varies, so regular monitoring is beneficial for personalized treatment.

When ABOi-KT recipients receive immune induction with RTX before surgery, the treatment plan is adjusted by monitoring the proportion of CD19^+^ B cell changes. For patients with a ratio of CD19^+^ B cells between approximately 10%–15%, calculated on the basis of a patient’s body surface area of 1.73 m^2^, using RTX 100 mg at 4 weeks, 2 weeks, and 24 h before surgery is recommended. When the proportion of CD19^+^ B cells is ≥ 15%, using RTX 200, 100, and 100 mg at 4 weeks, 2 weeks, and 24h before surgery, respectively, are recommended. When the proportion of CD19^+^ B cells is ≤10%, using RTX 100 mg at 4 and 2 weeks before surgery is recommended. Moreover, children and recipients with low body weight should reduce the dosage as deemed appropriate by the clinician (23, 24). Research has observed that ABOi-KT recipients using RTX still maintain a low B cell level within 6 months following surgery and gradually recover thereafter (25). Our conclusion is consistent with this. Our study shows that the B cell count in the KT group was significantly lower than that in the healthy control group, MN group and pemphigus group before treatment. At 4 weeks, 8 weeks, 12 weeks, 16 weeks, and 20 weeks after RTX treatment, more than 94.4%, 81.9%, 92.1%, 76.9%, and 88.5% of KT recipients maintained relatively low levels of B cell count and percentage. After 24 weeks, the regeneration rate of B cell counts and percentage in KT recipients gradually increased, reaching 63.3% and 67.3%. It was indicating that B cells were effectively suppressed during the treatment period, and the clearance effect of RTX remained consistently effective. At 20 weeks following RTX treatment, the number and percentage of B cells in one KT recipient showed a significant increase, indicating individual differences in B cell regeneration after RTX induction treatment in kidney transplant recipients. Peripheral blood B cell monitoring helps to personalize the evaluation of RTX treatment efficacy and timely detect rejection reaction occurrence and the timing of re-treatment.

The Remuzzi team conducted a study on the RTX treatment regimen for patients with MN by monitoring B cells and administered RTX (375 mg/m^2^). After one dose of treatment, peripheral blood B cells were completely cleared and began to recover from the third month, returning to the normal range from 6 to 11 months (26, 27). Most of the present study found that in patients with MN, B cells levels remained low for 20 weeks following RTX treatment. Our research indicates that before RTX treatment, the count and percentage of the B cells in MN group were significantly lower than those in pemphigus group, and remained significantly lower after treatment. Within 4 weeks after RTX treatment, there was no regeneration of B cells in MN patients, and the B cell levels gradually recovered after 24 weeks, which was consistent with previous studies. 71.8% (with a count increase of over 10 cells/uL) and 59.4% (with a percentage increase of over 1%) of patients had already started high-level expression of B cells around 24 weeks, and the B regeneration of such patients was approximately between 16 and 24 weeks. Thus, regular monitoring can help identify cases of B cell regeneration and provide timely re-treatment. However, this study also noted that some patients with MN (Case 2) had already shown a significant increase in the number and proportion of peripheral blood B cells after 15 weeks of treatment, with a doubling increase observed by 19 weeks following treatment. Individual differences were observed in the timing of B cell regeneration in patients with MN. Moreover, increasing the monitoring frequency can help detect disease recurrence and progression early on and help patients enter the next cycle of treatment in a timely manner, thus facilitating long-term effective control of the condition.

Kanwar evaluated the efficacy of different doses of RTX treatment regimens in patients with pemphigus and observed that although no statistically significant difference was noted in the time required to reach the treatment endpoint between the high-dose (RTX 1,000 mg twice a week) and low-dose groups (RTX 500 mg twice a week), monitoring CD19^+^ B lymphocytes revealed that the high-dose group maintained a longer B cell clearance status (12 weeks in the high-dose group *vs.* 4 weeks in the low-dose group), and recurrence was less common and had a longer duration in the low-dose group (28). In recent study showed that there existed a large number of B, T lymphocytes and plasma cells in the skin lesions in patients with pemphigus (29, 30). The study showed that there were far more the fraction of CD19+ B cells and antigen-specific B cells in pemphigus lesions than in perilesional skin and peripheral blood. However, there were very few B cells in nonlesion samples, and Dsg-specific B cells were barely detectable. Furthermore, many abundant clones of skin B cells connect with low-abundance clones of peripheral B cells, which indicates that some abundant skin B cell clones might migrate to the peripheral blood. The data from the SCID xenograft mice and BCR sequencing together indicate that lesional Dsg-specific B cells might circulate among lymph nodes, peripheral blood and pemphigus lesions (30). Our study is consistent with this. Our study shows that patients with pemphigus had significantly higher pretreatment B cell levels than those with MN, KT and healthy adults, and a trend of being higher than those in patients with NMO was also observed. Patients with pemphigus had the highest incidence of regeneration after 24 weeks of treatment, reaching 86.6% (with a count increase of over 10 cells/uL) and 69.5% (with a percentage increase of over 1%). Moreover, at the first follow-up 4 weeks following treatment, the B cell expression level was higher than that in the other disease groups. This suggests that to achieve a more thorough, effective, and long-lasting inhibitory effect on B cells and reduce the disease recurrence rate, patients with pemphigus must select a high RTX treatment dose. Additionally, it suggests that regular B cell monitoring is particularly significant for individualized treatment and prevention of disease recurrence in patients with pemphigus.

Early studies have suggested that patients with NMO should receive repeated RTX treatment when CD19^+^ B cells can be detected in peripheral blood or every 6–12 months (31, 32). However, some studies have noted that for patients with NMO, repeated treatment with RTX every 6–9 months is insufficient to prevent recurrence in each patient (33), which may be related to the significant individual differences in B cell regeneration after using RTX in patients with NMO. In another study, 17% of patients repopulated B cells prior to 6 months. During this repopulation patients are at risk for relapse and their disability is directly tied to the number and severity of relapses (20). Studies have shown that most clinical relapses occur following memory B cell regeneration, and once the number of B cells reaches 1%, it will quickly exceed 2% within a few days (33, 34). The Diagnosis and Treatment Guidelines for Optic Neuromyelitis Spectrum Disorders recommend monitoring peripheral blood B lymphocyte subsets in patients with NMO receiving RTX treatment: When the proportion of CD19^+^ or CD20^+^ B cells is >1% or the proportion of CD27^+^ memory B lymphocytes is >0.05%, repeating RTX treatment is recommended (16). Our research indicates that the probability of B cell regeneration in NMO patients within 4 weeks was less than 2%, and gradually increased thereafter. Patients with NMO had a higher incidence of B cell regeneration than those in other disease groups at 20 weeks. After 24 weeks of RTX treatment, 64.3% of patients with NMO had an increase in B cell count exceeding 10 cells/uL, while 52.4% of patients had an increase in B cell percentage exceeding 1%. Adjusting the time interval for patients to receive treatment on the basis of the monitoring results will help improve efficacy, reduce potential side effects, and save medical costs (19). This study observed that case 4 had already shown B cell regeneration during the first follow-up 10 weeks following treatment. These research results suggest that the monitoring time of peripheral blood B cells in patients with NMO treated with RTX should be moved forward, and the monitoring frequency should be appropriately increased to help timely evaluate the effectiveness of B cell clearance treatment, detect the timing of patients needing repeat RTX treatment earlier and more effectively, and improve treatment effectiveness.

When RTX is used for treating B-lymphocytic non-Hodgkin’s lymphoma, tracking and testing every 3 months within 6 months following treatment and follow-up every 6 months after 6 months of the treatment is recommended (35). The frequency of follow-up after treatment for children and adolescents with aggressive mature B cell non-Hodgkin’s lymphoma typically starts at 1 month following discontinuation of medication, then every 3–6 months from, then annually, and continues until 5 years following diagnosis (36). To date, no established standard for the starting time, monitoring frequency, and cut-off point of peripheral blood B cell testing is available for patients with autoimmune diseases and kidney transplant recipients receiving RTX treatment. Our research indicates that KT recipients and pemphigus patients experienced regeneration every month. More than 76.9% of KT recipients maintained low levels of expression within 20 weeks after treatment. Meanwhile, for patients with pemphigus, over 73.9% of them didn’t experience B cell regeneration within 20 weeks after treatment. In addition, MN patients didn’t show B cell regeneration within 4 weeks, and less than 2% of NMO patients experienced rebound within 4 weeks. Between 8 and 20 weeks, about 64% -95.6% of MN patients had low levels of B cells, while 7.69%-32% of patients with NMO experienced rebound. Thus, peripheral blood B cell monitoring in patients is beneficial for the timely understanding of the patient’s immune status, evaluating the treatment efficacy, better elucidating the timing of RTX retreatment, and guiding individualized clinical treatment, thereby improving the long-term treatment effect and quality of life of patients. Therefore, regular peripheral blood B cell monitoring at the beginning and after treatment is recommended. For KT recipients and pemphigus patients, the first follow-up should be conducted within 4 weeks after RTX treatment, followed by B cell monitoring every 4 weeks thereafter. For MN patients, the first follow-up should be conducted within 6-8 weeks after RTX treatment, followed by B cell monitoring every 4 weeks thereafter for the patients with MN or NMO. Due to 32.4% of NMO patients not monitoring B cells within 4 weeks, further research is needed to determine whether it is recommended to postpone the first B cell monitoring to 6-8 weeks. And the optimal approach is to conduct B cell subset testing concurrently. The decision whether to repeat RTX treatment or adjust other treatment plans is made on the basis of clinical evaluation and the degree of recovery in the number or proportion of B cells.

Plasmablasts are plasma cell precursors differentiated from activated B cells, with some forming short-lived plasma cells and others forming long-lived plasma cells, which survive in the body for a long time, leading to disease recurrence and difficulty in treatment. The differentiation of plasma cells is tightly regulated by intrinsic transcription factors and the surrounding microenvironment (37), and is a coordinated process with both genetic and epigenetic regulation (38). Although plasmablasts don’t express CD20, RTX can deplete the precursor cells of plasmablasts population, thereby reducing the number of plasmablasts and achieving the goal of relieving the disease. Therefore, its level can reflect the efficacy and disease control status of RTX. However, some patients still maintained high levels of plasmablasts expression after anti-CD20 treatment, mainly because traditional B cell deletion therapy is ineffective against long-lived plasma cells which were already present in the patient’s body (39). So that using anti-CD38 therapy is another option for patients with poor efficacy after RTX treatment and patients with high levels of plasmablasts expression.

## 5 Conclusions

The expression level of B cells in patients with pemphigus was the highest than the patients with other diseases before and after RTX treatment. After treatment with rituximab, the B cell count of most patients significantly decreased to below 10 cells/uL. Patients with pemphigus or KT recipients showed B cell regeneration within 4 weeks after treatment, and thereafter experienced regeneration every month. Therefore, it is recommended to monitor KT recipients or patients with pemphigus every 4 weeks. MN patients did not show B cell regeneration within 4 weeks, while NMO patients had a B cell regeneration probability of less than 2% within 4 weeks. Therefore, it is recommended to undergo the first follow-up within 6-8 weeks after RTX treatment for the patients with MN, followed by B cell monitoring every 4 weeks thereafter for the patients with MN or NMO. Due to the fact that 32.4% of NMO patients did not monitor B cells within 4 weeks, whether it is recommended to delay the first B cell regeneration monitoring to 6-8 weeks still needs further verification. The vast majority of patients’ B cell regeneration occured after 24 weeks of treatment. However, there were still some patients with low levels of B cells who may experience disease recurrence, so plasmablast monitoring is also recommonded.

## Data Availability

The raw data supporting the conclusions of this article will be made available by the authors, without undue reservation.
